# Clostridium difficile suppresses colonic vasoactive intestinal peptide associated with altered motility

**DOI:** 10.1155/S096293519500072X

**Published:** 1995-11

**Authors:** A. Nassif, W. E. Longo, R. Sexe, M. Stratton, J. Standeven, A. M. Vernava, D. L. Kaminski

**Affiliations:** Department of Surgery The Surgical Research Institute St. Louis University School of Medicine St. Louis MO USA

## Abstract

We investigated whether *Clostridium difficile* toxin alters colonic tissue levels of vasoactive intestinal peptide (VIP) at the expense of changes in colonic motility in the isolated perfused rabbit left colon. Colonic inflammation was induced by the intracolonic administration of 10^−8^ M C. *difflcile* toxin. Strain gauge transducers were sewn onto the serosal surface of the colon to evaluate colonic motility. C. *difflcile* administration produced histologic changes consistent with epithelial damage. This was associated with an increased production of prostaglandin E_2_ and thromboxane B_2_. Tissue levels of VIP but not substance P were significantly reduced. This was associated with an increased number of contractions per minute and an average force of each colonic contraction. These results suggest that tissue levels of VIP are suppressed by C. *difflcile* and may participate in colonic dysmotility during active inflammation.


Mediators of Inflammation 4, 452–453 (1995)

### Clostridium difficile suppresses colonic vasoactive intestinal peptide associated with altered motility

A. Nassif, W. E. Longo,[CA] R. Sexe, M. Stratton, J. Standeven, A. M. Vernava and D. L. Kaminski

Department of Surgery, The Surgical Research Institute, St. Louis University School of Medicine, St. Louis, MO, USA

[CA]Corresponding Author

**WE investigated whether *Clostridium difficile* toxin alters colonic tissue levels of vasoactive intestinal peptide (VIP) at the expense of changes in colonic motility in the isolated perfused rabbit left colon. Colonic inflammation was induced by the intracolonic administration of $10^{-8}$M *C. difficile* toxin. Strain gauge transducers were sewn onto the serosal surface of the colon to evaluate colonic motility. *C. difficile* administration produced histologic changes consistent with epithelial damage. This was associated with an increased production of prostaglandin $E_2$ and thromboxane $B_2$. Tissue levels of VIP but not substance P were significantly reduced. This was associated with an increased number of contractions per minute and an average force of each colonic contraction. These results suggest that tissue levels of VIP are suppressed by *C. difficile* and may participate in colonic dysmotility during active inflammation.**

**Key words:** *Clostridium difficile*, motility, prostanoids, vasoactive intestinal peptide

### Introduction

*Clostridium difficile* toxin is the causative agent of pseudomembranous colitis. This disease entity is associated with watery diarrhoea and altered motility, and is often effectively treated with enteral antibiotics.[1] Colonic neuropeptides such as VIP and substance P are abundant in the gut and maintain various physiological functions, such as regulation of colonic motility. We have previously demonstrated that platelet-activating factor (PAF) increases colonic tissue levels of VIP and substance P which were inhibited by a specific PAF antagonist.[2] In contrast, in further studies we found that trinitrobenzene (TNB) suppresses levels of these neuropeptides, which were ameliorated by neural blockade.[3] The aim of the present study was to evaluate the possible role of endogenous VIP and substance P in the dysmotility changes of the left colon of the rabbit to *Clostridium difficile* toxin.

### Materials and Methods

The experimental procedure for producing colonic inflammation by other agents known to induce colitis in the isolated rabbit colon has been reported previously.[4] After isolating the colon, it was placed on a temperature controlled base, humidified whole organ perfusion apparatus (Mx International, Aurora, CO) and allowed to equilibrate with an intra-arterial Kreb's Ringers bicarbonate (KRB) infusion for the first 30 min period. This was followed by the intraluminal infusion of $10^{-8}$M *C. difficile* toxin while continuing intra-arterial infusion of KRB for 30 min. At the end of this period, *C. difficile* was discontinued and the colon was allowed to recover for 30 min, infusing only KRB intra-arterially. In a separate set of experiments, KRB was infused intraluminally during the entire 90 min period. Strain gauge transducers were sewn onto the serosal surface of the colon to measure colonic motility. Motility was calculated as four indices: (1) contractions/min; (2) peak force per contraction measured in grams; (3) average force per contraction; and (4) the minute motility index (MMI) calculated as the sum of the contractions, weighed by the peak force, and expressed as a per minute average. At the end of the experiment, tissue samples of colon were taken and immersed in liquid nitrogen and then stored at −70°C. A second section of colon was taken and evaluated histologically to determine the extent of inflammation. Tissue levels of prostaglandin $E_2$ ($PGE_2$), thromboxane $B_2$ ($TxB_2$), VIP and substance P were determined by well described radioimmunoassays.[3,5]

### Results

No colitis was observed in KRB treated colons. *C. difficile* treated colons demonstrated evidence of inflammation that was confined to the mucosa

© 1995 Rapid Science Publishers

**Table 1.** The effect of *C. difficile* on tissue $PGE_2$, $TxB_2$, VIP, substance P and colonic motility

| | KRB ($n$=4) | *C. difficile* ($n$=4) |
|---|---|---|
| Tissue VIP levels[a] | 221.2 ± 31.6 | 107.6 ± 16.4* |
| Tissue SP levels[a] | 164.6 ± 27.7 | 179.1 ± 13.5 |
| Tissue $PGE_2$ levels | 25.2 ± 6.4 | 174.8 ± 31.9* |
| Tissue $TxB_2$ levels | 18.7 ± 5.4 | 32.4 ± 8.8* |
| Contractions/min | 10.9 ± 3.5 | 14.7 ± 3.3* |
| Peak force[b] | 2.5 ± 0.11 | 1.9 ± 0.09 |
| Average force[c] | 0.44 ± 0.3 | 0.72 ± 0.08* |
| MMI[d] | 15.1 ± 3.5 | 16.2 ± 4.1 |

*$p < 0.05$, *C. difficile* and KRB.
[a]pg/mg wet tissue.
[b]Peak force per contraction.
[c]Average force per contraction.
[d]Minute Motility Index.

and submucosa and did not involve the muscularis propria. Tissue concentrations of $PGE_2$ and $TxB_2$ in *C. difficile* treated colons were significantly increased compared to KRB treated colons. Tissue levels of VIP were significantly suppressed while levels of substance P remained unchanged in *C. difficile* treated colons. Motility results demonstrated that *C. difficile* increased both the number of contractions per min and the average force per contraction (Table 1).

### Discussion

Our results demonstrate that *C. difficile* toxin produces a significant inflammatory response and altered colonic motility that correlates with alterations in tissue levels of VIP. A recent study[6] utilizing *C. difficile* toxin A on rabbit distal colonic loops demonstrated an increased number of spike bursts without altering slow-wave frequency that was associated with increased production of prostanoids and leukotrienes. The authors concluded that this result may be indirect resulting from the production of motility altering arachidonic acid metabolites. The results were similar to that seen by Gilbert[7] who demonstrated that similar alterations in ileal circular smooth muscle myoelectric and contractile activity were seen with toxin A.

Other investigators, as well as our own, have demonstrated that disturbances in colonic motility may be related to fluctuating levels of IL-1, PAF and eicosanoids in both TNB and PAF models of colonic inflammation.[3,8–10] We speculate from our previous studies involving alterations in colonic neuropeptides that disturbance in colonic motility may be related to a mechanism involving arachidonic acid metabolites released during inflammation and neuropeptide fluctuations. Presently, our data suggest that VIP is suppressed by *C. difficile* and may participate in colonic dysmotility disturbances during inflammatory states of the left colon. Studies are in progress to determine whether neural blockade or pretreatment with synthetic VIP antagonists antagonize the changes in neuropeptide release, inflammation and motility changes seen by *C. difficile*.

### References

1. Larson AE, Price AB. Pseudomembranous colitis: presence of Clostridial toxin. *Lancet* 1977; **2:** 1312–1324.
2. Deshpande Y, Longo WE, Chandel B, *et al.* Effect of platelet-activating factor (PAF) and its antagonists on colonic dysmotility and tissue levels of colonic neuropeptides. *Eur J Pharmacol* 1994; **256:** 121–123.
3. Sexe R, Stratton M, Nassif A, Kaminski DL, Longo WE. Lidocaine ameliorates trinitrobenzene sulfonic acid suppression of tissue levels of colonic neuropeptides. *Surg Research Comm* (in press).
4. Chandel B, Niehoff M, Deshpande Y, Standeven J, Vernava AM, Kaminski DL, Longo WE. The isolated perfused colon: a novel model to study colonic inflammation and motility. *Surg Research Comm* 1994; **16:** 125–129.
5. Kaminski DL, Andrus CH, German D, Deshpande YG. The role of prostanoids in the production of acute acalculous cholecystitis by platelet-activating factor (PAF). *Ann Surg* 1990; **212:** 455–461.
6. Burakokk R, Zhao L, Celifarco AJ, Rose KL, Donovan V, Pothoulakis C, Percy WH. Effects of purified *Clostridium difficile* toxin A on rabbit distal colon. *Gastroenterology* 1995; **109:** 348–354.
7. Gilbert R, Triadafilopoulos G, Pothoulakis C, Giampaolo C, Lamont JT. Effect of purified *C. difficile* toxin on intestinal smooth muscle. *Am J Physiol* 1989; **256:** G759–G766.
8. Pons L, Droy-Lefaix MT, Bveno L. Leukotriene D4 participates in colonic transit disturbances induced by intracolonic administration of trinitrobenzene sulfonic acid in rats. *Gastroenterology* 1992; **102:** 149–156.
9. Morteav O, More J, Pons L, Lveno L. Platelet-activating factor and interleukin-1 are involved in colonic dysmotility in experimental colitis in rats. *Gastroenterology* 1993; **104:** 47–56.
10. Longo WE, Standeven J, Chandel B, Deshpande Y, Vernava AM, Kaminski DL. Platelet-activating factor mediates trinitrobenzene induced dysmotility in the left colon. *Mediators of Inflammation* 1995; **4:** 17–18.

ACKNOWLEDGEMENT. This study was supported by USPHS Grant DK 27695.

**Received 22 August 1995; accepted 28 August 1995**
